# Phytochemicals and Bioactivities of the Halophyte Sea Mayweed (*Tripleurospermum maritimum* L.)

**DOI:** 10.3390/md23110420

**Published:** 2025-10-30

**Authors:** Clément Lemoine, Maria João Rodrigues, Xavier Dauvergne, Stéphane Cérantola, Luísa Margarida Batista Custódio, Christian Magné

**Affiliations:** 1 Laboratoire Géoarchitecture: Territoires, Urbanisation, Biodiversité, Environnement, Université de Brest, CS 93837, F-29200 Brest, Francexavier.dauvergne@univ-brest.fr (X.D.); 2 Centre of Marine Sciences, Faculty of Sciences and Technology, University of Algarve, Ed. 7, Campus of Gambelas, 8005-139 Faro, Portugal; mjrodrigues@ualg.pt (M.J.R.); lcustodio@ualg.pt (L.M.B.C.); 3 Service Général des Plateformes Technologiques, Plateforme RMN-RPE, Université de Bretagne Occidentale, 6 Avenue V. le Gorgeu, CS 93837, F-29238 Brest, France; stephane.cerantola@univ-brest.fr

**Keywords:** antidiabetic, anti-inflammatory, antioxidant, bio-guiding, NMR characterization, *Tripleurospermum maritimum*, whitening activity

## Abstract

Sea mayweed (*Tripleurospermum maritimum* L. syn. *Matricaria maritima*) is a halophytic species widely distributed along the Atlantic shoreline. Unlike other *Tripleurospermum* species, the chemical composition and biological activities of this halophyte have received no attention. Here, a hydroalcoholic extract of sea mayweed leaves was evaluated for in vitro antioxidant (DPPH, ABTS, and FRAP bioassays), anti-inflammatory (NO reduction in RAW 264.7 macrophages), anti-diabetic (alpha-glucosidase inhibition), neuroprotective (inhibition of acetylcholinesterase), and skin protective (tyrosinase, melanogenesis, elastase, and collagenase inhibition) activities. Solid–liquid partition chromatography of the extract and NMR characterization of its fractions allowed the identification of some major compounds, including fructo-oligosaccharides in the MeOH_20%_ fraction, a new carbohydrate called tripleurospermine (1), 3-5-dicaffeoylquinic acid (2) in the MeOH_40%_ fraction, and matricaria lactone (3) in the MeOH_80%_ fraction. MeOH_40_ fraction exhibited strong antioxidant, anti-tyrosinase (thus skin-whitening potential), and anti-glycosidase activities (anti-diabetic potential), whereas MeOH_80%_ fraction showed anti-inflammatory and anti-diabetic potential. Overall, our results suggest that sea mayweed may have dietary or medicinal uses due to its biochemical composition and bioactivities.

## 1. Introduction

The *Matricaria* and *Tripleurospermum* genera have long been considered synonymous within the *Asteraceae* (*Compositae*) family. Recently, they have been separated into two closely related taxa in the *Anthemideae* tribe [1,2]. Together, these two genera consist of about 40 species widely distributed in Europe, North Africa, America, and temperate Asia. *Matricaria* species are often used for ornamental, medicinal, or feeding purposes, whereas *Tripleurospermum* do not have uses yet. Over the past decade, many pharmacological properties have been described in the *Matricaria* genus, including anti-inflammatory [3,4], anti-tumoral, antioxidant, neuroprotective [5], anti-allergic [6], antimicrobial [7,8], anticancer [9], sedative, stomachic, antispasmodic, and antidiarrheal activities [10].

So far, a number of studies have examined the chemical composition of some members of *Matricaria* or *Tripleurospermum*, especially of their essential oils [11,12]. In addition, valuable secondary metabolites such as phenolic compounds [13,14,15] and related coumarins [16,17], terpenes [18], and polyacetylenes were identified in *Matricaria* species (see [19] as a review). However, only a few *Tripleurospermum* species have been phytochemically investigated up until this point, including *T. auriculatum* or *T. disciforme* [20,21,22], whereas little or no information is available about most species, including *T. maritimum*. Therefore, with the aim of characterizing the chemical composition and bioactivities of this halophyte, we describe here the partial purification of hydroalcoholic leaf extract and the identification of some of its major components, along with several biological properties.

**Figure 1:**
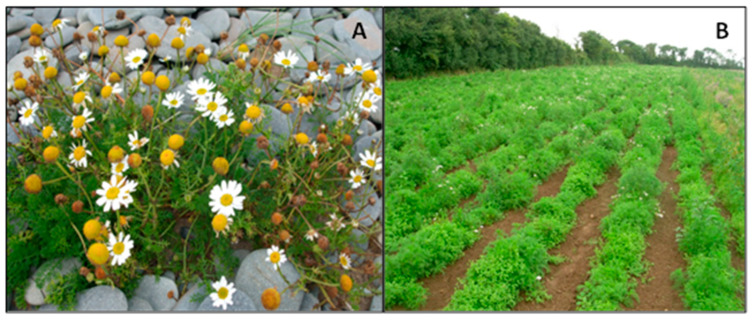
Plants of sea mayweed (*Tripleurospermum maritimum* L.) on maritime rocks (**A**) and grown in open field (**B**) (source: C. Magné).

*Tripleurospermum maritimum* (L.) W.D.J. Koch (previously *Matricaria maritima* L.) is a biennial halophytic plant. Commonly known as false mayweed or sea mayweed, it grows along the Atlantic coast of Europe, mostly on sandy beaches and pebbles. Alternatively, it can be easily grown in open fields through standard agronomic techniques (Figure 1), yielding about 1.2 TDW/ha (Magné, unpublished data).

We previously reported that sea mayweed leaves contain a large amount of fructo-oligosaccharides [23], which are the major components of a water extract. Since no other biochemicals have been reported hitherto in this species, we have investigated the biochemical composition of a polar extract of *T. maritimum*. We describe here the identification of several major constituents in leaf hydro-ethanolic extract and evaluate in vitro several biological activities of nutraceutical and cosmetic interest in this extract and its fractions.

## 2. Results

### 2.1. Total Phenolic Content

**Figure 2:**
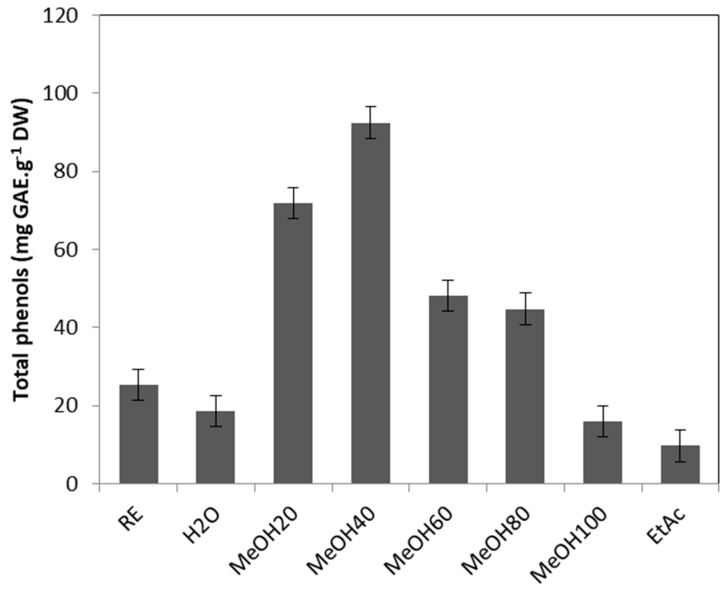
Total phenolic content (mg GAE/g DW) of *T. maritimum* raw extract and fractions. RE: Raw extract; H_2_O: Water fraction; MeOH20: MeOH_20%_ fraction; MeOH40: MeOH_40%_ fraction; MeOH60: MeOH_60%_ fraction; MeOH80: MeOH_80%_ fraction; MeOH100: MeOH_100%_ fraction; EtAc: Ethyl acetate fraction. Means ± standard deviations of three replicates are represented, and different letters above the bars indicate significantly different means (*p* < 0.05).

The crude extract of *Tripleurospermum maritimum* leaves exhibited a high phenolic amount, with 22.1 mg GAE/g DW (Figure 2). As the onset of a bioguided fractionation of this extract aimed at identifying the major components of the raw extract, phenolics were detected mainly in the fractions eluted with 20, 40, 60, and 80% methanol, which exhibited 2.85, 3.7, 1.9, and 1.8 times more phenolic compounds than the crude extract, respectively.

### 2.2. Antioxidant Activities

**Figure 3:**
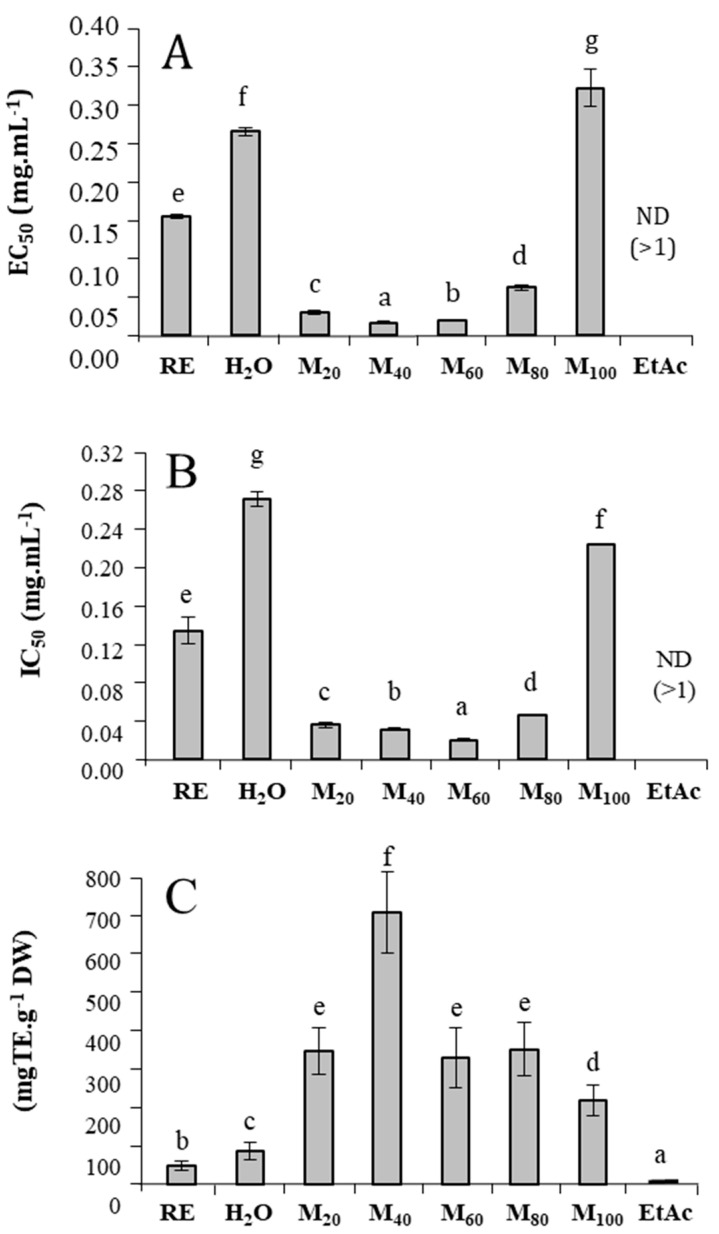
Antioxidant activities of *T. maritimum* raw extract and its fractions. (**A**) ferric reducing capacity (EC_50_ in mg/mL); (**B**) radical scavenging activity against DPPH (IC_50_ in mg/mL); (**C**) radical scavenging activity against ABTS (mg TE/g DW). RE: Raw extract; H_2_O: Water fraction; M_20_: MeOH_20%_ fraction; M_40_: MeOH_40%_ fraction; M_60_: MeOH_60%_ fraction; M_80_: MeOH_80%_ fraction; M_100_: MeOH_100%_ fraction; EtAc: Ethyl acetate fraction. Means ± standard deviations of three replicates are represented, and different letters above the bars indicate significantly different means (*p* < 0.05).

The crude extract of *T. maritimum* exhibited high reducing activity, with an EC_50_ value of 0.155 mg/mL for the FRAP bioassay (Figure 3A). After fractionating the extract, the MeOH_20%_, MeOH_40%_, MeOH_60%_, and MeOH_80%_ fractions exhibited the highest reducing capacity, with EC_50_ values 5, 8.9, 7.5, and 2.6 times lower than that of the crude extract, respectively. The same trend was observed in the DPPH assay, where MeOH_20%_, MeOH_40%_, MeOH_60%_, and MeOH_80%_ fractions showed 3.9, 4.4, 6.5, and 3 times lower IC_50_ values than that of the crude extract (0.116 mg/mL), respectively (Figure 3B). Similarly, the ABTS radical scavenging capacity was distributed among the fractions eluted with methanol solutions. Thus, MeOH_20%_, MeOH_40%_, MeOH_60%_, MeOH_80%_, and MeOH_100%_ fractions exhibited 7.2, 14.8, 6.85, 7.3, and 4.5 times higher activities than the crude extract (55.3 mg TE/g DW), respectively (Figure 3C). Conversely, the last fraction (eluted with ethyl acetate) showed negligible or no activities using every antioxidant bioassay.

### 2.3. Other Biological Activities

#### 2.3.1. Anti-Ageing Activity

Sea mayweed crude extract exhibited an appreciable activity against tyrosinase, with a value of 234 mg KAE/g DW. Of the seven fractions eluted from this extract, the last five exhibited a significantly higher activity than that of the crude extract (*p* < 0.05) (Table 1). Among them, the MeOH_100%_ and ethyl acetate fractions were the most active, with around 700 mg KAE/g DW. Moreover, a significant anti-melanogenic action on B16 4A5 melanoma cells was found in the last fraction (eluted with ethyl acetate), being 50% more active than the standard (arbutin). However, neither the raw extract nor its fractions exhibited any activities against melanogenesis or other ageing-related enzymes like elastase and collagenase until a concentration of 0.5 g/L.

#### 2.3.2. Anti-Inflammatory Activity

Sea mayweed raw extract did not show any capacity to inhibit NO production by RAW 264.7 macrophages. However, two of its fractions (namely MeOH_60%_ and MeOH_80%_) were able to strongly reduce this indicator of inflammation (Table 1). Accordingly, fractions eluted with 60% and 80% MeOH inhibited NO production five and three times more intensely than the positive control L-NAME (27.81 μg/mL), respectively.

#### 2.3.3. Neuroprotective Activity

Sea mayweed extract and fractions were evaluated for their capacity to inhibit AChE, the primary enzyme responsible for the hydrolytic metabolism of the neurotransmitter acetylcholine. However, only the MeOH_60%_ fraction exhibited a slight inhibitory effect on AChE (Table 1).

#### 2.3.4. Anti-Diabetic and Anti-Obesity Activities

Pancreatic lipase is the enzyme responsible for the digestion and absorption of triglycerides, and its inhibition is one of the most widely studied methods to determine the potential activity of natural products to prevent and treat obesity. Here, neither extracts nor fractions showed inhibitory activity on rat lipase enzyme in vitro. Then, their capacity to inhibit α-amylase and α-glucosidase was assessed. Although sea mayweed raw extract was inactive on these enzymes, four fractions strongly inhibited α-glucosidase: MeOH_40%_, MeOH_60%_, MeOH_80%_, and ethyl acetate fractions (Table 1). Accordingly, these four fractions exhibited a remarkable inhibitory effect, with 15-, 50-, 6-, and 150-fold lower IC_50_ values than the standard glucosidase inhibitor acarbose. In addition, no amylase inhibition could be detected in these fractions.

### 2.4. Structural Determination

**Figure 4:**
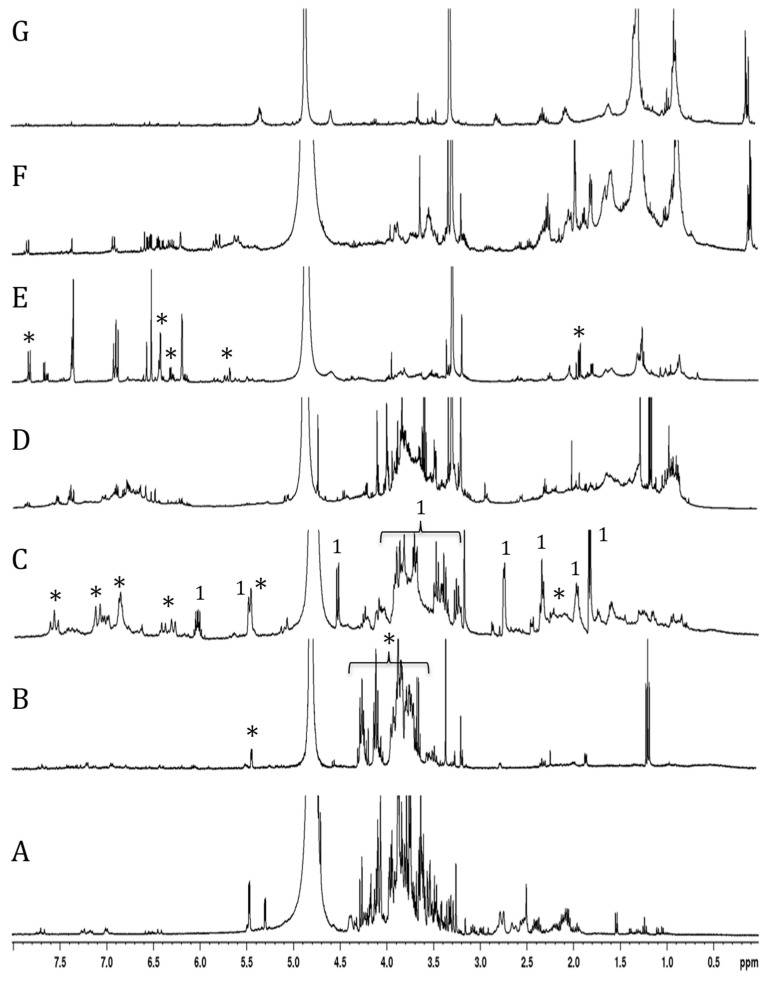
H^1^ NMR spectra of *T. maritimum* extract fractions eluted with water (**A**), 20% MeOH (**B**), 40% MeOH (**C**), 60% MeOH (**D**), 80% MeOH (**E**), 100% MeOH (**F**), and ethyl acetate (**G**). Stars in spectra (**B**,**C**,**E**) indicate signals of fructo-oligosaccharide, 3,5-dicaffeoylquinic acid, and matricaria lactone, respectively. Specific signals (1) of tripleurospermine are indicated on spectrum (**C**).

Following purification of the extract, the characterization of the active fractions was performed by NMR spectroscopy. The ^1^H-NMR spectrum of the water fraction (effluent) mainly showed the presence of numerous signals from oses (including specific signals of glucose and sucrose between 4.8 and 5.5 ppm) and amino acids (mainly alanine, proline, and glutamine between 1.5 and 2.5 ppm) (Figure 4A). Major signals in the 3.6–4.3 ppm range, assigned to fructo-oligosaccharides, were found in the MeOH_20%_ fraction (Figure 4B).

**Figure 5:**
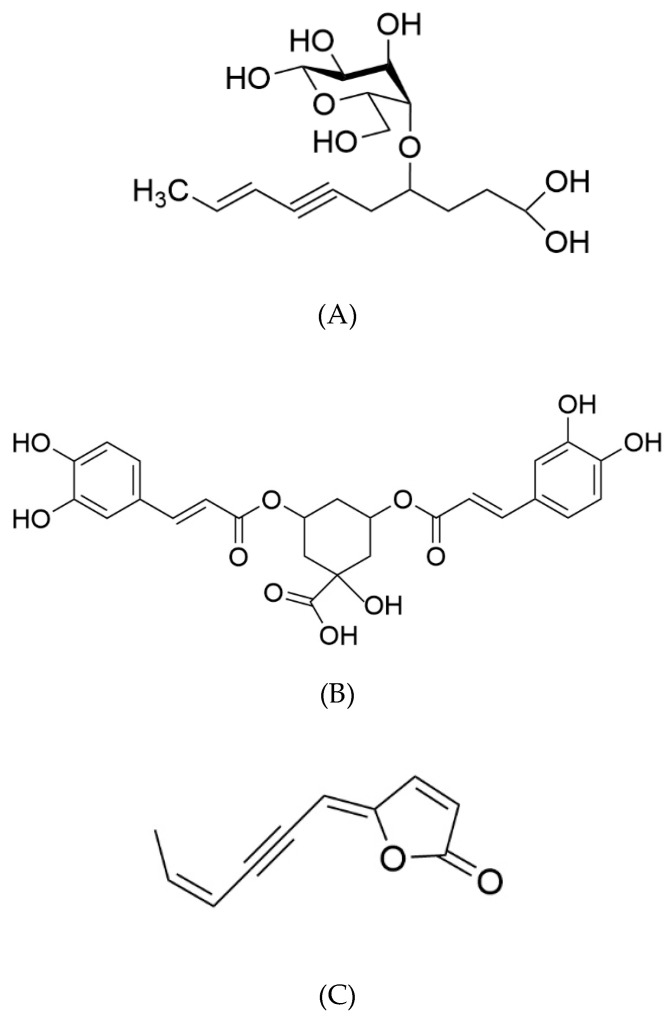
Chemical structures of major compounds identified in *T. maritimum* extract fractions: tripleurospermine (**A**), 3,5-di-O-caffeoylquinic acid (**B**), and matricaria lactone (**C**).

The ^1^H-NMR spectrum of the MeOH_40%_ fraction exhibited numerous signals in the aliphatic (1.8–2.8 ppm), anomeric (3.2–4.6 ppm), and unsaturated (5.5–7.6 ppm) regions (Figure 4C). Further examination of this fraction, following sub-fractionation and subsequent ^13^C- and 2D NMR analyses, allowed us to identify a major compound in the most polar sub-fraction, eluted with 20% MeOH. This compound was characterized by signals between 3.2 and 4.2 ppm associated with a doublet in the anomeric region (4.5 ppm), suggesting a sugar residue. Other signals in the unsaturated (5.5 and 6.1 ppm) and aliphatic (from 1.8 to 2.8 ppm) regions were also found, and 2D-NMR COSY and TOCSY experiments showed two spin systems (Supplementary Data S3). The first one was characterized by signals at 4.56 (H1, d), 3.29 (H2, dd), 3.47 (H3, m), 3.39 (H4, m), 3.44 (H5, m), 3.71 (H6a, m), and 3.90 (H6b, m) ppm. Corresponding carbons were identified by means of an HMQC experiment at 105.4, 76.1, 78.6, 72.5, 78.5, and 63.8 ppm, respectively (Supplementary Data S2). The second spin system was characterized by signals at 1.85 (H1′, dd), 6.06 (H2a′, m), 5.49 (H3′, dd), 2.76 (H6′, d), 3.92 (H7′), 1.98 (H8′, m), and 2.32 (H9′, t) ppm. These protons could be correlated by the HMQC experiment to carbons at 18.5, 142.5, 111.9, 28.4, 82.1, 32.9, and 36.1 ppm, respectively. Examination of the J-MOD spectrum suggested that the first spin system belongs to a sugar residue, and HMBC experiments allowed us to correlate this residue to the second spin system (Supplementary Data S3). Finally, acid hydrolysis of this subfraction provided a glucose residue, and 2D NMR experiments showed that the lateral chain corresponded to dec-8-ene-6-ynoic acid (Table 2). This observation was confirmed by mass spectrometry analysis, where the detected molecular ion [M+Na] corresponded well to the theoretical *m*/*z* for a C_6_H_12_O_6_Na_2_C_10_H_12_O_2_ structure (367.1663 versus 367.1354, respectively). Finally, the structure was identified as 4-*O*-β-D glucose derivative of dec-8-ene-6-ynoic acid (Figure 5A). This newly reported compound was named tripleurospermine. Moreover, MeOH_25%_ subfraction showed signals in the aliphatic (2.1–2.3 ppm), anomeric (3.6, 4.1 ppm), and aromatic (5.6, 6.3, 6.8–7.1, and 7.6 ppm) regions (already seen in Figure 4C). The latter group of signals may be assigned to the dicaffeoyl moiety. Following ^13^C- and 2D NMR experiments on this fraction (Supplementary Data S1), we identified unequivocally 3,5-di-*O*-caffeoylquinic acid as the major constituent (Figure 5B).

The MeOH_60%_ fraction showed several signals in the 6–8 ppm region, corresponding to aromatic compounds (Figure 4D). Some of them are likely glycosylated, as revealed by the signals in the ose and anomeric proton regions.

The NMR spectrum of the MeOH_80%_ fraction showed numerous signals between 5.6 and 8 ppm, and pronounced ones between 1 and 2 ppm, corresponding to aromatic and aliphatic protons, respectively (Figure 4E). Further fractioning of this fraction allowed us to isolate a pure compound eluted with 65% MeOH. This constituent was characterized by signals at 1.9, 5.7, 6.1, 6.3, and 7.9 ppm on the ^1^H-NMR spectrum, and 2D-NMR (COSY, HMQC, and HMBC) experiments allowed us to identify this compound as 5-(4-hexen-2-ynylidene) furan-2-one, also known as matricaria lactone (Figure 5C).

The last two fractions, eluted with MeOH_100%_ and ethyl acetate, contained apolar compounds showing aliphatic protons and minor signals in the 1–2 ppm and 6–7 ppm regions, respectively (Figure 4F,G).

## 3. Discussion

Over the past decades, the search for natural products in plants has led to the discovery of a number of biologically active substances, particularly secondary metabolites. These compounds are widely represented in most medicinal or halophytic species, where they exhibit a number of biological activities [24]. Within Asteraceae, the chemical composition and biological properties of extracts from *Aster* or *Matricaria* genera are widely documented [10,15,19,25] and mainly reveal antioxidant properties. However, research on *Tripleurospermum* has received little attention [20,26]. Therefore, with the aim of characterizing the chemical composition and biological activities of the halophyte sea mayweed (*T. maritimum*), we describe the partial purification of hydroalcoholic leaf extract and the identification of some of its major components, along with several biological properties.

Crude extract of *T. maritimum* leaves showed a medium content of total phenolic compounds, compared to other halophytic species. It was lower than well-known rich plants like *Limonium vulgare*, *Mesembryanthemum edule*, and *Arthrocnemum macrostachyum* [24,27,28]. However, its content was similar to that in *Crithmum maritimum* [29] and higher than in *Juncus acutus*, *Halimione portulacoides, Limoniastrum monopetalum*, or *Halocnemum strobilaceum* [28,30].

Solid–liquid partition chromatography of the crude extract of sea mayweed leaves on C18-bound silica gel yielded fractions enriched in some major constituents. The fraction eluted with 20% methanol (MeOH_20%_ fraction) appeared to be composed mainly of an oligofructan, *α*-D-Glc*p*-(1 → 2)-[*β*-D-Fru*f*-(2 → 1)-*β*-D-Fruc*f*]_n_-(2 → 1)-*β*-D-Fru*f*, which is already described in wild *Matricaria maritima* leaf extract [23]. Along with well-known prebiotic uses, this kind of fructo-oligosaccharide (FOS) may have promising pharmacological applications or be used for reinforcement of polymer matrix composites thanks to its mechanical properties [31]. Here, this fraction exhibited a mild antioxidant capacity, probably related to its low phenolic content. However, this fraction did not show any other biological activity with the bioassays used here.

Particular attention was given to the MeOH_40%_ fraction, where 1D and 2D NMR studies led to the identification of 3,5-dicaffeoylquinic acid and a never-before-reported carbohydrate, called tripleurospermine, as major constituents. Interestingly, this fraction exhibited the highest phenolic content and the strongest antioxidant activities (with FRAP and ABTS bioassays). Phenolic compounds are well known to have a number of biological properties, including antioxidant activity [32]. Consequently, halophytes, which are rich in phenolics, have been reported to have a strong antioxidant potential [24,33,34]. Among phenolic compounds, caffeoyl derivatives are known to contribute significantly to the antioxidant activity of plants such as Asteraceae [25]. These authors reported the presence of 3,5-dicaffeoylquinic acid in several members of this family, including a species closely related to *T. maritima*, *Matricaria perforata*. The antioxidant capacity of the two main compounds identified in the MeOH_40%_ fraction has been evaluated in their respective enriched subfractions. Interestingly, our results suggested for the first time that the newly identified molecule, tripleurospermine, would exert a significant activity, though lower than that of the caffeoyl derivative. Importantly, the MeOH_40%_ fraction also exhibited a marked capacity to inhibit tyrosinase activity, a well-known oxidizing enzyme responsible for melanin formation in the skin. The activity level, reported here for the first time in this species, was close to that of the standard tyrosinase inhibitor kojic acid. Moreover, it appears higher than that recently reported in other halophytic species like *Citrullus colocynthis*, *Crithmum maritimum*, *Daemia cordata*, *Plantago coronopus*, or the Asteraceae *Inula crithmoides* [29,35,36]. In this family, Duke [27] reported such anti-tyrosinase property in the closely related species *Matricaria recutita*, thanks to its phenolic compounds kaempferol, *p*-coumaric acid, and quercetins. We finally confirmed these observations by measuring a significant anti-tyrosinase activity in the subfractions enriched with tripleurospermine or 3,5-dicaffeoylquinic acid. Therefore, these two compounds are likely responsible for the tyrosinase inhibition found in the MeOH_40%_ fraction. This result confirms that a positive correlation could be made between antioxidant and anti-tyrosinase activities, as reported previously by Choi et al. [37]. However, this fraction exhibited no activities against melanogenesis or other ageing-related enzymes like elastase and collagenase, confirming a previous observation made in *Matricaria recutita* [38]. In addition, the MeOH_40%_ fraction exhibited a marked capacity to inhibit α-glucosidase activity, a well-known hypoglycemic enzyme involved in carbohydrate digestion. This observation is in agreement with recent work by Spínola and Castilho [39], showing a strong hypoglycemic effect of caffeoylquinic acids from Asteraceae plants. Therefore, this fraction could be used as an adjuvant to prevent Type 2 diabetes.

The MeOH_60%_ fraction showed the widest range of biological activities, as seen by its antioxidant, anti-tyrosinase, anti-inflammatory, neuroprotective, and anti-diabetic capacities. Its strong capacity to inhibit NO production and α-glucosidase was noteworthy, making this fraction a potential source of bioactive drugs, particularly against inflammation or diabetes syndromes. Since the NMR spectrum of the MeOH_60%_ fraction showed the prevalence of phenolic compounds, it could be that these constituents are responsible for its wide range of activities. Accordingly, the anti-inflammatory effect of phenolics was reported [40,41], as well as their anti-diabetic properties in other Asteraceae [39,42,43]. In addition, phenolics have been shown to inhibit tyrosinase [44] and ameliorate the neurodegenerative process [45,46]. Further investigation aimed at purifying major constituents in this fraction is needed to ascribe the contribution of the different compounds in the exhibited activities.

The MeOH_80%_ fraction exhibited a strong antioxidant capacity. Following sub-fractioning, a major constituent was identified as 2(5H)-furanone, 5-(4-hexen-2-yn-1-ylidene), also known as matricaria lactone. This monoterpene lactone was previously identified in other members of Asteraceae, including *Conyza* (=*Erygeron*) and *Chamomilla* (=*Matricaria*) genera [14,47], where it was reported to exert antifungal properties [48,49]. Since matricaria lactone appeared as the main component in the MeOH_80%_ fraction, it likely explains its strong antioxidant and anti-tyrosinase properties. However, to the best of our knowledge, neither antioxidant nor anti-tyrosinase activity had been reported for this compound hitherto. Interestingly, the MeOH_80%_ fraction also showed a strong anti-inflammatory capacity (three-fold higher than the standard L-NAME). Since terpene lactones have been reported to have anti-inflammatory effects [42], it could be that the NO inhibitory effect of the MeOH_80%_ fraction is due to matricaria lactone. In addition, this fraction strongly inhibited α-glucosidase. A potential anti-diabetic action of lactones has not been reported hitherto, and further work is needed to assess the potential effect of pure matricaria lactone on this enzyme.

The last two fractions, eluted with MeOH_100%_ and ethyl acetate, mainly exhibited apolar compounds, as evidenced by their NMR analysis showing major signals of aliphatic protons. These signals suggest the presence of terpenoids and/or steroids as major constituents in these fractions, since such chemicals are commonly found in Asteraceae [49,50,51]. Interestingly, steroids and terpenoids have been reported to inhibit tyrosinase [52,53,54], and the last two fractions of sea mayweed extract exhibited the most powerful anti-tyrosinase activity, being as active as the whitening standard agent (kojic acid) used in the cosmetic industry. However, these fractions had low antioxidant activities, though tyrosinase catalyzed two oxidative reactions. Hence, the contribution of non-antioxidant mechanisms to tyrosinase inhibition could not be excluded. Moreover, the last fraction of sea mayweed extract was the only one to exhibit anti-melanogenesis properties and showed a remarkable α-glucosidase inhibitory action. These activities could be related to some low-polar constituents like terpenoids, as reported by Kanlayavattanakul and Lourith [44] and Patel et al. [55], respectively. In addition, no other skin anti-ageing activities (e.g., anti-elastase or anti-collagenase activities) were observed in sea mayweed raw extract or fractions at the tested concentrations. Our results confirm those reported in *Matricaria recutita*, a *T. maritima*-related species, where neither anti-elastase nor anti-collagenase activity could be found [38].

## 4. Materials and Methods

### 4.1. Chemicals, Culture Media, and Supplements

Lipase (EC 3.1.1.3), acetylcholinesterase (EC 3.1.1.7), α-glucosidase (EC 3.2.1.20), α-amylase (EC 3.2.1.1), tyrosinase (EC 1.14.18.1), and the B16 A45 murine melanoma cell line were purchased from Sigma-Aldrich (Hamburg, Germany). Folin-Ciocalteau phenol reagent, all chemicals (ABTS, L-DOPA, DPPH, DTNB, EGCG, and HEPES), standards (acarbose, arbutine, galantamine, and L-NAME), solvents (ethanol and methanol) used for chemical analyses, and bioassays were supplied by Sigma Aldrich (St. Louis, MO, USA).

### 4.2. Plant Material

Seeds of *T. maritimum* were collected on a sand beach at Le Conquet (48°21′37″ North, 4°46′15″ West, France). Seeds were disinfected with a saturated solution of calcium hypochlorite (30%) for 3 min and rinsed in distilled water. They were germinated for 10 days at 20 °C in Petri dishes on filter paper moistened with 0.1 mM CaSO_4_. Then, germinated seeds were transferred to 250 mL pots filled with a mixture of sand and sterile loam (1:1 *v*/*v*) and watered daily with Hewitt nutrient solution [56]. Seedlings were grown in a greenhouse under controlled conditions: 14–23 °C and 50–70% relative humidity, 8–16 h night–day photoperiod. The aerial parts of 3-month-old *T. maritimum* plants were harvested, immediately frozen, and freeze-dried. Dried samples were then powdered and stored at room temperature until extraction.

### 4.3. Extraction and Fractionation

About 1 g of leaf powder was homogenized with 25 mL of water/ethanol (1:2) under magnetic stirring at 4 °C for 20 min. This solvent is commonly considered an efficient means for the extraction of secondary metabolites, including phenolics, and it is suitable to provide extracts for food, cosmetic, or medicinal applications. After centrifugation of the mixture (15 min at 4 °C, 4.000× *g*), the resulting pellet was extracted twice following the same protocol. The supernatants were collected, pooled, and filtrated over glass wool. The obtained extract was concentrated by rotary evaporation at 40 °C and resuspended in 50% ethanol.

Fractionation of the raw extract (approx. 0.3 g) was performed by solid–liquid partition chromatography on C18-bound silica gel (GRACE Davisil RP18, Düren, Germany). The elution of polar compounds proceeded by increasing methanol concentration in water (successively 0, 20, 40, 60, 80, and 100%) and, finally, ethyl acetate. The fractions were then concentrated by rotary evaporation at 30 °C and resuspended in the corresponding solvent. Further fractionation of the MeOH_40_ fraction was performed on the same column, using the same method but with smaller MeOH increments (i.e., MeOH 20%, 25%, 30%, 35%, and 40%). When necessary, a subfraction was submitted to acid hydrolysis treatment (1 N HCl, 110 °C for 1 h) before structural elucidation with NMR experiments. All these fractions were used for the following bioassays.

### 4.4. Evaluation of Antioxidant Activities

#### 4.4.1. DPPH Scavenging Activity

The scavenging activity of the stable 1,1-diphenyl-2-picrylhydrazyl (DPPH) free radical was determined by the method of Marwah et al. [57]. Briefly, the reaction medium contained 100 μL of 100 μM DPPH solution in ethanol and 100 μL of plant extract at different concentrations (or water for the control). The reaction mixture was incubated in the dark for 15 min, and the absorbance was recorded at 517 nm with a Multiskan FC microplate reader (Thermo Scientific Technologies, Beijing, China). The assay was carried out in triplicate. The decrease in absorbance upon the addition of test samples was used to calculate the inhibition percentage (%IP) of DPPH radical, following the equation as follows:

%IP = [(A_c_ − A_s_)/A_c_] × 100 (1)

where A_c_ and A_s_ are the absorbances of the control and the test sample, respectively. From a plot of concentration against %IP, a linear regression analysis was performed to determine the antiradical activity, as expressed by the IC_50_ (extract concentration resulting in a 50% inhibition) value for each sample.

#### 4.4.2. Ferric Reducing Ability (FRAP)

The assay is based on the reaction of Fe^2+^ with 2,4,6-tri(pyridyl)-*s*-triazine (TPTZ) to form a violet-blue colour with maximal absorbance at 593 nm [58,59]. The FRAP solution was prepared by mixing 10 volumes of acetate buffer (300 mM, pH 3.6) with 1 volume of TPTZ (40 mM in HCl) and 1 volume of ferric chloride (20 mM in water). The solution was prepared daily and warmed at 37 °C for 10 min before use. A 280 μL aliquot of this solution was mixed with 20 μL of samples (extract, fractions, or water for the blank) in a 96-well microplate. The mixture was incubated at 37 °C in the dark for 30 min and then read at 593 nm. The increase in absorbance upon addition of test samples was used to calculate the reducing capacity, as expressed by the efficacy percentage (%EP):

%EP = [(A_s_ − A_c_)/A_c_] × 100 (2)

where A_s_ and A_c_ are the absorbances of the control and the test sample, respectively. From a plot of concentration against %EP, a linear regression analysis was performed to determine the EC_50_ (extract concentration resulting in 50% efficacy) value for each sample.

#### 4.4.3. ABTS Scavenging Activity

The ABTS radical scavenging assay was based on the method described by Re et al. [60] with a slight modification. Briefly, a 7 mM ABTS stock solution was prepared by dissolving ABTS in ethanol:water (5:1 *v*/*v*). Then, an aliquot of this solution was reacted with 2.45 mM potassium persulfate in ethanol:water (1:3 *v*/*v*) and allowed to stand in the dark at room temperature for 16–20 h to prepare the ABTS radical cation (ABTS^+^). This ABTS radical solution was diluted to an absorbance at 734 nm of 0.70 ± 0.02. Finally, the absorbance of a mixture consisting of the sample (or water for the blank) and ABTS reagent was measured at 734 nm. The antiradical capacity of the samples was expressed as Trolox equivalents (mg TE/g DW).

### 4.5. Evaluation of Anti-Ageing Activity

Anti-tyrosinase assay was performed using L-DOPA as substrate, according to the method described by Masuda et al. [61]. The samples (plant extract or fractions) were dissolved in 50% dimethyl sulfoxide (DMSO). Then, 40 μL of each sample was mixed with 80 μL of phosphate buffer (0.1 M, pH 6.8), 40 μL of tyrosinase (31 units/mL in phosphate buffer, pH 6.5), and 40 μL of 2.5 mM L-DOPA in a 96-well microplate. The absorbance of the mixture was measured at 475 nm and compared to that of a positive control containing kojic acid and a blank containing all the components except L-DOPA. The anti-tyrosinase activity was expressed as kojic acid equivalents (mg KAE/g DW).

Anti-elastase activity was assayed according to the method described by Kim et al. [62], slightly modified to perform the assay in 96-well microplates. The substrate N-Succinyl-Ala-Ala- Ala-p-nitroanilide (SANA) was dissolved in Tris-HCL buffer (0.2 M, pH 8.0) at 1.6 mM. Plant extract or fractions were incubated with porcine pancreatic elastase (E.C. 3.4.21.36) at 10 μg/mL for 20 min at 37 °C before adding substrate. Epigallocatechin gallate (EGCG) was used as a positive control. Absorbance at 410 nm was measured 20 min after substrate addition. The percentage of enzyme inhibition was calculated as follows:

Enzyme inhibition (%) = [(A_control_ − A_sample_)/A_control_] × 100 (3)

Anti-collagenase activity was assayed using the ENZO MMP-1 colorimetric kit (Enzo Life Sciences, Lyon, France), adapted to 96-well microplates. The assay was performed in 50 mM HEPES buffer (pH 7.5) containing 0.05% Brij-35, 1 mM DTNB, and 10 mM CaCl_2_. MMP-1 (Matrix MetalloProteinase-1) was dissolved in this buffer at an initial concentration of 0.76 units/mL. The synthetic substrate (chromogenic peptidic substrate) was dissolved in HEPES buffer to 1 mM. Plant extracts were incubated with the enzyme in buffer for 15 min before adding the substrate. The specific inhibitor [N-Isobutyl-N-(4-methoxyphenylsulfonyl) glycyl hydroxamic acid] was used as a positive control. Absorbance at 412 nm was measured every minute from 10 to 20 min after substrate addition. The percentage of enzyme inhibition was calculated as follows:

Enzyme inhibition (%) = (V_sample_/V_control_) × 100 (4)

where V_sample_ and V_control_ are the linear regression slopes of the inhibition due to sample and control, respectively.

Moreover, the anti-melanogenic activity of sea mayweed extract and fractions was assessed in vitro on B16 4A5 melanoma cells according to Bouzaiene et al. [63]. Cells were seeded at 3.5 × 10^4^ cells/well into 12-well plates, allowed to adhere for 24 h, and then treated with sea mayweed extract concentrations that allowed cellular viability higher than 80% for 72 h. Thereafter, adherent cells were trypsinized and solubilized in 1 mL of 1% sodium dodecyl sulphate (SDS). The absorbance of the samples was measured at 475 nm, and the melanin content was estimated using a standard curve of synthetic melanin (0–25 μg/mL).

### 4.6. Evaluation of Neuroprotective Activity

The neuroprotective property of sea mayweed extract was evaluated through the in vitro inhibition of acetylcholinesterase (AChE) according to Custódio et al. [64]. Samples (20 µL at concentrations of 1, 5, and 10 mg/mL) were mixed with 140 µL of sodium phosphate buffer (0.1 mM, pH 8.0) and 20 µL of AChE solution (0.28 U/mL) in 96-well microplates. The mixture was incubated for 15 min at room temperature, and the reaction was initiated by the addition of 10 µL of 4 mg/mL ATChI and 20 µL of 1.2 mg/mL DTNB. The absorbance was read at 405 nm, and the results were expressed as IC_50_ relative to a control containing water instead of extract. Galantamine was used as a positive control.

### 4.7. Evaluation of Anti-Diabetic Activity

Sea mayweed extract and fractions, at concentrations ranging from 1 to 5 mg/mL, were evaluated for their capacity to inhibit α-amylase and α-glucosidase according to Zengin [65]. Moreover, extracts and fractions were tested against porcine lipase according to McDougall et al. [66]. Acarbose was used as a positive control for α-amylase and α-glucosidase, and orlistat was used as a positive control for lipase inhibition. Results were expressed as IC_50_ relative to a control containing DMSO.

### 4.8. Evaluation of Anti-Inflammatory Activity

Nitric oxide (NO) production by LPS-stimulated RAW 264.7 macrophages was assessed as described by Rodrigues et al. [28]. RAW 264.7 cells were cultured in RPMI 1640 culture medium, enriched with 10% heat-inactivated FBS, 1% L-glutamine (2 mM), and 1% penicillin (50 U/mL)/streptomycin (50 μg/mL), and kept at 37 °C in a 5% CO_2_ humidified atmosphere. Murine cells were seeded in a 96-well plate at 2.5 × 10^5^ cells per well and allowed to adhere overnight. Then, they were co-treated with 100 ng/mL of LPS and sea mayweed raw extract (at concentrations that allowed cellular viability higher than 80%) for 24 h. NO production was assessed using the Griess assay. Results were expressed as a percentage of inhibition of NO production, relative to a control containing DMSO (0.5%, *v*/*v*), and compared to the positive control L-nitroarginine methyl ester (L-NAME).

### 4.9. NMR Analyses

Dry fractions were dissolved in 700 μL of 99.5% D_2_O and placed in 5 mm NMR tubes. ^1^H and ^13^C NMR spectra were obtained at 298°K on a Bruker DRX-400 spectrometer (400 MHz), equipped with a 5 mm dual ^1^H/^13^C probe head, using standard pulse sequences. A typical 1D ^1^H NMR spectrum consisted of 32 scans, and 2,2,3,3-tetradeuterio-3-(trimethylsilyl)-propanoic acid sodium salt was used as an internal standard. For ^13^C (J-mod) and 2D ^1^H NMR (COSY, HMBC, HMQC, and TOCSY) analyses, experiments were carried out at 298°K on a Bruker Avance III HD500 spectrometer equipped with an inverse 5 mm TCI cryoprobe with z gradient. Data were processed using TopSpin^®^ 4.0 software (Bruker, Wissembourg, France).

### 4.10. Mass Spectrometry Analysis

When needed, characterization of a purified compound was performed on an Autoflex (Bruker, France) ion trap mass spectrometer equipped with a Matrix-Assisted Laser Desorption/Ionization (MALDI) source and a quadrupole time-of-flight (QTOF) analyzer. Spectra were acquired in negative-ion mode over a mass range from *m*/*z* 100 to 1500 with 5 Hz frequency. Operating parameters of the MALDI ion source were as follows: capillary voltage 3 kV, dry gas flow 6 L/min, dry gas temperature 200 °C, nebulizer pressure 0.7 bar, collision radio frequency 700.0 V, transfer time 100.0 μs, and pre-pulse storage 7.0 μs. Ultrapure nitrogen was used as drying and nebulizer gas, and argon was used as collision gas. Collision energy was set automatically from 15 to 75 eV depending on the *m*/*z* of the fragmented ion. Acquired data were calibrated internally with sodium formate introduced to the ion source at the beginning and ending of each separation via a 20 μL loop. Control and data acquisition were carried out with the Bruker DataAnalysis 4.3 software.

### 4.11. Statistical Analyses

All extractions and assays were conducted in triplicate. Results were expressed as mean ± standard deviation (SD), and the means were compared by using one-way analysis of variance (ANOVA) followed by Duncan’s multiple range tests performed by the “Statistica v. 5.1” software (Statsoft, 2008). Differences between individual means were deemed to be significant at *p* < 0.05. The IC_50_ values were calculated by the sigmoidal fitting of the data using the GraphPad Prism v. 5.0 programme.

## 5. Conclusions

Biological activities and chemical composition of *Tripleurospermum maritimum* were thoroughly investigated here for the first time. Solid–liquid partition chromatography of the crude hydroalcoholic extract and NMR analyses allowed us to identify fructo-oligosaccharides (FOS) in the MeOH_20%_ fraction, a new carbohydrate tripleurospermine (1), 3-5-dicaffeoylquinic acid (2) in MeOH_40%_ fraction, and matricaria lactone (3) in the MeOH_80%_ fraction as the major components. Though the crude extract of *T. maritimum* leaves did not show strong biological activities in vitro, some of its fractions revealed highly promising potential. Thus, MeOH_40%_ fraction exhibited strong antioxidant, anti-tyrosinase (thus skin-whitening potential), and anti-glycosidase activities, but no other anti-ageing properties. The MeOH_60%_ fraction exhibited the highest diversity of activities, with antioxidant, anti-tyrosinase, and remarkable anti-inflammatory, neuroprotective, and anti-diabetic properties. The MeOH_80%_ fraction showed anti-inflammatory and anti-diabetic potential. The less-polar fractions were particularly promising with skin-whitening potential. Overall, this work shows that sea mayweed could find dietary or medicinal uses, as an interesting source of prebiotics (FOS) and of phyto-pharmaceutical preparations for skin-ageing, inflammation or diabetes control, and neuroprotection. Further work should be addressed (i) to characterize the strongly bioactive MeOH_60%_ fraction, (ii) to confirm the biological activities of pure tripleurospermine and of matricaria lactone, and (iii) to validate in vivo the results observed here. Alternatively, investigations on seasonal and organ variability of biochemical contents and bioactivities in *T. maritimum* plants grown in an open field should also be addressed.

## Figures and Tables

**Table 1:** Skin-whitening (tyrosinase inhibition), anti-inflammatory (inhibition of NO production in RAW 264.7 macrophages), anti-melanogenic (inhibition of melanin production in RAW 264.7 macrophages), neuroprotective (acetylcholine esterase inhibition), and anti-diabetic (alpha-glucosidase inhibition) activities of sea mayweed polar extract and its fractions. Means ± SEM of three replicates are presented, and different letters indicate significantly different means (*p* < 0.05). ND: not determined.

	Tyrosinase Inhibition (mgKAE/g DW)	Melanogenesis Inhibition	NO Inhibition	AchE Inhibition	Anti-α Glucosidase
		(% at 125 μg/mL)	(IC_50_, μg/mL)	(IC_50_, mg/mL)	(IC_50_, mg/mL)
Raw extract	294.29 ± 49.56 c	ND	ND	ND	ND
MeOH_20%_	178.42 ± 26.98 d	ND	ND	ND	ND
MeOH_40%_	452.59 ± 72.73 b	ND	ND	ND	0.20 ± 0.01 c
MeOH_60%_	527.89 ± 15.65 b	ND	5.69 ± 0.30 a	0.06 ± 0.00 b	0.02 ± 0.00 a
MeOH_80%_	472.92 ± 42.70 b	ND	8.63 ± 0.55 a	ND	0.57 ± 0.04 d
MeOH_100%_	707.52 ± 59.97 a	ND	ND	ND	ND
EtAc	719.67 ± 53.45 a	23.77 ± 1.17 b	103.17 ± 2.08 c	ND	0.06 ± 0.02 b
Arbutin		15.95 ± 0.50 a			
L-NAME			27.81 ± 1.93 b		
Galantamine				0.01 ± 0.00 a	
Acarbose					3.14 ± 0.09 e

**Table 2:** 1D- and 2D-NMR data (in ppm) of tripleurospermine identified in the MeOH_40%_ fraction of *T. maritimum* leaf extract.

N°	^1^H-NMR	^13^C-NMR, Type	TOCSY	HMBC
>Aglycon				
1	-	185.7, qC	-	-
2	2.32 (t; 7.6)	36.1, CH_2_	H-3; 4; 5	C-1; 3; 4
3	1.98 m	32.9, CH_2_	H-2; 4; 5	C-1; 2; 5; 6
4	3.92	82.1, CH	H-2; 3; 5; H-1′	C-1
5	2.76 (d; 3.8)	28.4, CH_2_	H-2; 3; 4; 8; 10	C-3; 4; 6; 8; 9; 10
6	-	94.1, qC	-	-
7	-	82.4, qC	-	-
8	5.49 (dd; 11.1)	112, CH	H-4; 5; 9; 10	C-6; 10
9	6.06 (m; 7.1)	142.5, CH	H-5; 8; 10	C-7; 8; 10
10	1.85 (dd; 7.1)	18.5, CH_3_	H	C-7; 8; 9
>O-β-D-Glucopyranoside				
1′	4.56 (d; 7.9)	105.4, CH	H-2′; 3′; 4′; 5′; 6′	C-2′; C-4
2′	3.29 (dd; 9.25)	76.1, CH	H-1′; 3′; 4′; 5′; 6′	C-1′; 3′
3′	3.47 m	78.6, CH	H-1′; 2′; 4′; 5′; 6′	C-1′; 2′; 4′
4′	3.39 m	72.5, CH	H-1′; 2′; 3′; 5′; 6′	C-3′; 5′; 6′
5′	3.44 m	78.5, CH	H-1′; 2′; 3′; 4′; 6′	C-1′; 2′; 4′, 6′
6′	3.90 m; 3.71 m	63.8, CH_2_	H-1′; 2′; 3′; 4′; 5′	C-1′; 2′; 5′

## Data Availability

The original contributions presented in this study are included in the article/Supplementary Materials. Further inquiries can be directed to the corresponding author.

## Supplementary Materials

The following supporting information can be downloaded at https://www.mdpi.com/article/10.3390/md23110420/s1; Supplementary data S1: NMR spectroscopic data (500 MHz, CDCl3) for 3,5-di-O-caffeoylquinic acid identified in MeOH40% fraction of *T. maritimum* leaf extract. (s: singlet, d: doublet, t: triplet, dd: doublet of doublets, m: multiplet, brs: broad singlet). Supplementary data S2: NMR spectroscopic data (500 MHz, CDCl3) for tripleurospermine identified in MeOH_40%_ fraction of *T. maritimum* leaf extract. (s: singlet, d: doublet, t: triplet, dd: doublet of doublets, m: multiplet). Supplementary data S3: 2D-NMR spectra of MeOH_20%_ sub-fraction of the MeOH_40%_ fraction of *T. maritimum* leaf extract, showing spin systems of tripleurospermine in COSY (A), HMQC (B) and HMBC (C) experiments.

## Abbreviations

ABTS: 2,2′-azino-bis(3-ethylbenzothiazoline-6-sulphonic acid); L-DOPA: L-dihydroxyphenylalanine; DPPH: 2,2-diphenyl-1-picrylhydrazyl; DTNB: 5,5′-dithiobis-(2-nitrobenzoic acid); EGCG: Epigallocatechin gallate; FRAP: Ferric reducing antioxidant power; HEPES: N-2-Hydroxyethylpiperazine-N′-2-ethanesulfonic acid; HMBC: heteronuclear multiple bond coherence; HMQC: heteronuclear multiple quantum coherence; MALDI: Matrix-Assisted Laser Desorption/Ionization; MeOD: deuterated methanol; MeOH: methanol; NMR: nuclear magnetic resonance.
