# Supplementary material for: Phytochemicals and Bioactivities of the Halophyte Sea Mayweed (Tripleurospermum maritimum L.)
Source: Mar Drugs. 2025 Oct 30;23(11):420. doi: 10.3390/md23110420 (PMC12654308; doi:10.3390/md23110420)

Supplementary data

Supplementary data S1: NMR spectroscopic data (500 MHz, $CDCl_3$) for 3,5-di-*O*-caffeoylquinic acid identified in $MeOH_{40\%}$ fraction of *T. maritimum* leaf extract.
(s: singlet, d: doublet, t: triplet, dd: doublet of doublets, m: multiplet, brs: broad singlet).

^{1}H-NMR (500 MHz, MeOH-d4) δ (ppm) : 2.35 - 2.13 (4H, m, H-2, 6), 5.45 - 5.36 (2H, m, H-3/H-5), 3.96 (1H, dd, J = 9.9, 3.4 Hz, H-4), 7.06 (2H, brs, H2, 2'), 6.77 (2H, dd, J = 7.8, 1.2 Hz, H-5/H-5'), 6.97, 6.96 (2H, dd, J = 8.2, 2.0 Hz, H-6,6'), 7.60, 7.57 (1H each, d, J = 15.9 Hz, (H-7/H-7'), 6.35, 6.26 (1H each, d, J = 15.9 Hz, H-8-8').

^{13}C-NMR (100 MHz, MeOH-d4) δ (ppm): 74.7 (C-1), 36.0 (C-2), 72.5 (C-3), 70.5 (C-4), 72.1 (C-5), 38.6 (C-6), 177.3 (COOH), 127.9, 127.8 (C-1, 1'), 115.1 (C-2, 2'), 146.8 (C-3, 3'), 149.6, 149.5 (C-4, 4'), 116.5, 116.5 (C-5, 5'), 123.1, 123.0 (C-6, 6'), 147.3, 147.0 (C-7, 7'), 115.5, 115.2 (C-8, 8') 168.9, 168.3 (C-9,9').

Supplementary data S2: NMR spectroscopic data (500 MHz, $CDCl_3$) for tripleurospermine identified in $MeOH_{40\%}$ fraction of *T. maritimum* leaf extract. (s: singlet, d: doublet, t: triplet, dd: doublet of doublets, m: multiplet).

N°	^{1}H-NMR	^{13}C-NMR, type	TOCSY	HMBC
> Aglycon				
1	-	185.7, qC	-	-
2	2.32 (t; 7.6)	36.1, CH_2	H-3 ; 4 ; 5	C-1 ; 3 ; 4
3	1.98 m	32.9, CH_2	H-2 ; 4 ; 5	C-1 ; 2 ; 5 ; 6
4	3.92	82.1, CH	H-2 ; 3 ; 5 ; H-1’	C-1
5	2.76 (d; 3.8)	28.4 CH_2	H-2 ; 3 ; 4 ; 8 ; 10	C-3 ; 4 ; 6 ; 8 ; 9 ; 10
6	-	94.1, qC	-	-
7	-	82.4, qC	-	-
8	5.49 (dd; 11.1)	112, CH	H-4 ; 5 ; 9 ; 10	C-6 ; 10
9	6.06 (m; 7.1)	142.5, CH	H-5 ; 8 ; 10	C-7 ; 8 ; 10
10	1.85 (dd; 7.1)	18.5, CH_3	H	C-7 ; 8 ; 9
> O-*β*-D-Glucopyranoside				
1’	4.56 (d; 7.9)	105.4, CH	H-2’ ; 3’ ; 4’ ; 5’ ; 6’	C-2’ ; C-4
2’	3.29 (dd; 9.25)	76.1, CH	H-1’ ; 3’ ; 4’ ; 5’ ; 6’	C-1’ ; 3’
3’	3.47 m	78.6, CH	H-1’ ; 2’ ; 4’ ; 5’ ; 6’	C-1’ ; 2’ ; 4’
4’	3.39 m	72.5, CH	H-1’ ; 2’ ; 3’ ; 5’ ; 6’	C-3’ ; 5’ ; 6’
5’	3.44 m	78.5, CH	H-1’ ; 2’ ; 3’ ; 4’ ; 6’	C-1’ ; 2’ ; 4’, 6’
6’	3.90 m; 3.71 m	63.8, CH_2	H-1’ ; 2’ ; 3’ ; 4’ ; 5’	C-1’ ; 2’ ; 5’

Supplementary data S3: 2D-NMR spectra of $MeOH_{20}$ sub-fraction of the $MeOH_{40\%}$ fraction of *T. maritimum* leaf extract, showing spin systems of tripleurospermine in COSY (A), HMQC (B) and HMBC (C) experiments.

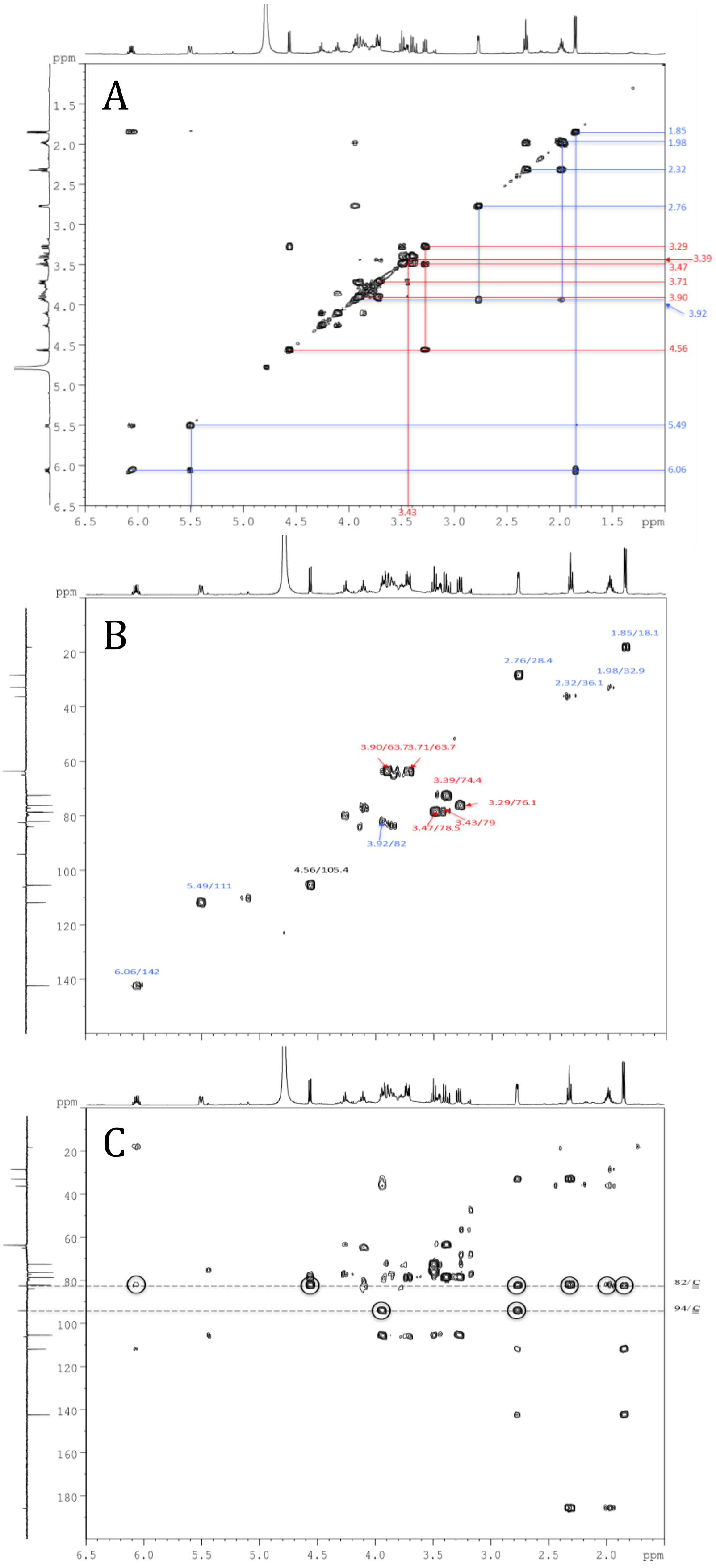

Supplement: Supplementary file 1 [file marinedrugs-23-00420-s001.zip › marinedrugs-3866125-supplementary.pdf]
